# Metabolomic profiling of Wilson disease, an inherited disorder of copper metabolism, and diseases with similar symptoms but normal copper metabolism

**DOI:** 10.1186/s13023-023-02900-5

**Published:** 2023-09-11

**Authors:** Yijie Qiu, Mingchuan Su, Xina Xiao, Dingzi Zhou, Linshen Xie

**Affiliations:** 1 West China School of Public Health and West China Fourth Hospital, Sichuan University, Chengdu, 610041 China; 2 West China-PUMC C.C. Chen Institute of Health, Sichuan University, Chengdu, 610041 China

**Keywords:** Copper, Wilson’s disease, Liver cirrhosis, Parkinson’s disease, Metabolomic profile, Lipidomics

## Abstract

**Background:**

Wilson’s disease (WD) is a hereditary disorder that results in the accumulation of copper. The pathogenic mechanism is not well understood, and diagnosing the disease can be challenging, as it shares similarities with more prevalent conditions. To explore the metabolomic features of WD and differentiate it from other diseases related to copper metabolism, we conducted targeted and untargeted metabolomic profiling using ultra-high-performance liquid chromatography-tandem mass spectrometry (UPLC-MS/MS) and liquid chromatography-tandem mass spectrometry (LC-MS). We compared the metabolomic profiles of two subgroups of WD patients, namely hepatic WD (H-WD) and neurological WD (N-WD), H-WD patients and liver cirrhosis patients (who exhibit similar symptoms but have normal copper levels), and N-WD patients and Parkinson’s disease patients (who exhibit similar symptoms but have normal copper levels).

**Results:**

Our pairwise comparisons revealed distinct metabolomic profiles for male and female WD patients, H-WD and N-WD patients, N-WD and Parkinson’s disease patients, and H-WD and liver cirrhosis patients. We then employed logistic regression analysis, receiver operating characteristic (ROC) analysis, and model construction to identify candidate diagnostic biomarkers that differentiate H-WD from liver cirrhosis and N-WD from Parkinson’s disease. Based on the spatial distribution of data obtained via PLS-DA analysis, we discovered variations in hydrophilic metabolites (aminoacyl-tRNA biosynthesis; alanine, aspartate, and glutamate metabolism; phenylalanine metabolism; arginine biosynthesis; and nicotinate and nicotinamide) and lipophilic metabolites (TG(triglyceride) (16:0_16:1_22:6), TG (16:0_16:0_22:6), and TG (16:0_16:1_22:5)) between H-WD and N-WD. Moreover, WD patients display metabolic traits that distinguish it from comparable conditions (liver cirrhosis and Parkinson’s disease).

**Conclusions:**

Our analysis reveals significant variations in the levels of metabolites in critical metabolic pathways and numerous lipids in WD.ROC analysis indicates that three metabolites may be considered as candidate biomarkers for diagnosing WD.

## Introduction

Wilson’s disease (WD), also named hepatolenticular degeneration, is a rare inherited disorder that leads to copper accumulation [1]. This disease is caused by homozygous mutations or compound heterozygous mutations (two different mutant alleles) in the *ATP7B* gene, which encodes a transmembrane copper transport ATPase. Specific mutations of *ATP7B* that cause impaired copper homeostasis and copper overload in the liver, brain, and other organs are responsible for this disease [2].Patients with WD typically experience hepatic symptoms, neuropsychiatric symptoms, and symptoms in other organs. Misdiagnosis and delayed treatment may occur due to the clinical manifestations of WD being similar to other common diseases not related to copper metabolism, such as hepatitis, liver cirrhosis, and Parkinson’s disease [3]. Although liver damage and nervous system disorders are common manifestations of WD, some patients may only develop liver damageor nervous system disorders. The mechanisms underlying the diverse clinical manifestations of WD are not yet fully understood, but some studies suggest that lipid oxidation of biological membranes, and excessive production of oxygen free radicals may be responsible for the cytotoxic effects associated with this condition [4].

Copper plays a multifacetedrole in human metabolism, It serves as a cofactor for a variety of key metabolic enzymes that drive a broad range of physiological processes [4, 5]. A recent publication in the journal Science has shed light on a new form of cell death caused by copper accumulation, which has been termed cuproptosis. This novel cell death pathway is regulated by mitochondrial ferredoxin 1-mediated protein lipoylation [6]. Thus, metabolomic study of Wilson’s disease, a condition arising from disrupted copper metabolism, can facilitate the detection of metabolite perturbations resulting from copper accumulation and enhance comprehension of the exact mechanisms underlying this novel pathway of cell death.

Diagnosing WD can often prove challenging due to the variability of its clinical presentation, which may manifest as asymptomatic biochemical abnormalities, acute liver failure, or neurological symptoms. Given the absence of a singular, highly sensitive and specific diagnostic test, an accurate diagnosis requires a comprehensive evaluation utilizing a combination of clinical, biochemical, and genetic testing modalities [7]. Metabolomics is a powerful tool for detecting differences between normal and diseased metabolism by analyzing metabolites and identifying those associated with disease. These metabolites can be used as biomarkers for diagnosing, classifying, treating, and predicting the prognosis of various diseases. Metabolomics also enables the study of interactions between different metabolic pathways, shedding light on the mechanisms and biological processes that underlie disease development. By applying metabolomics technology, we can gain deeper understanding of the metabolic characteristics of WD disease, discover novel biomarkers, and offer effective diagnostic recommendations [8].

According to convention, two distinct methodologies are employed in the field of metabolomics: targeted metabolomics and untargeted metabolomics. Targeted metabolomics involves the quantification of specific groups of chemically characterized and annotated metabolites, while untargeted metabolomics entails the comprehensive analysis of all measurable analytes in a sample, including unknown chemicals. [9, 10].

In this study, we utilized untargeted metabolomics alongside targeted lipidomic approach to differentiate between the two primary clinical subtypes of WD: hepatic WD (H-WD) and neurologic WD(N-WD). Additionally, we also compared the metabolomic profiles of WD patients with the those of individuals afflicted with Parkinson’s disease and liver cirrhosis, two kinds of diseases that exhibit similar symptoms but are not caused by copper accumulation.

## Materials and methods

### Patient

There were 34 WD patients (24 N-WD and 10 H-WD), 11 patients with liver cirrhosis, and 14 patients with Parkinson’s disease. All patients were selected from the same hospital, and samples were collected from January 2020 to April 2022.In addition, medical history and severity of illness were taken into account in the selection of patients for inclusion, excluding those withcomorbidities such as cancer, severe heart failure, chronic kidney disease, strokeand alcoholic fatty liver disease.This study complies with the provisions of the Helsinki Declaration and was approved by the Medical Ethics Committee of West China Fourth Hospital (HXSY-EC-2,020,074).

### Untargeted metabolomics

A 100 µL sample of blood plasma from each patient was added into 100 µL of a mixed internal standard solution and 400 µL of methanol (− 20℃), vortexed for 60 s, centrifuged at 4℃ for 10 min at 12,000 rpm, and then the 500 µL supernatant was transferred into a2 mL centrifuge tube. Samples were then concentrated and dried using a vacuum. The dry sample was dissolved with 150 µL of an 80% methanol solution, centrifuged at 4℃ for 10 min at 12,000 rpm, and the supernatant was collected for liquid chromatography-mass spectroscopy (LC-MS) analysis. Chromatographic separation was performed using Thermo Vanquish system, which was equipped with an ACQUITY UPLC® HSS T3 column (150 × 2.1 mm, 1.8 μm, Waters) at 40 ℃.The temperature of the autosampler was 8 °C. The analyte was Gradient elution with 0.1% formic acid (A2) and 0.1% formic acid (B2) or 5 mM of ammonium formate (A3) and acetonitrile (B3) at a velocity of 0.25 mL/min. After balance, each sample was injected with 2 µL. The linear gradient of solvent B2/B3 (v/v) increased as follows: 0–1 min, 2% B2/B3; 1–9 min, 2–50% B2/B3; 9–12 min, 50–98% B2/B3; 12–13.5 min, 98% B2/B3; 13.5–14 min, 98–2% B2/B3; 14–20 min, 2% B2-positivemodel (14–17 min, 2% B3-negative model).The ESI-MS analysis were performed using Thermo Q Exactive mass spectrometer. Sheath gas and auxiliary gas were set to 30 and 10 arbitrary units respectively. The capillary temperatures was 325 °C. The analyser scanned over a mass range of m/z81-1,000forafull scan at a mass resolution of 70,000.MS/MS data-dependent acquisition (DDA) experiments were performed with a HCD scan. Anormalized collision energyof30Ev was used.

### Targeted metabolomics

A 50 µL sample of blood plasma was homogenized with 1mL of mixture of methanol, MTBE, and an internal standard mixture(the mix of samples). The solution was mixed for 15 min, 200 µL of water was added, the solution was mixed again for 1 min, and it was then centrifuged at 4℃ for 10 min at 12,000 rpm. Then, 500 µLofthe supernatant was concentratedand dried using aspeed vacuum concentrator.Anddissolvedried samplewith 200 µL of the mobile phase B, and then stored at − 80℃. Finally,thesolution was added into the sample bottle for LC-MS/MS analysis.Thesampleswere analyzed using an LC-ESI-MS/MS system (UPLC, ExionLC AD, https://sciex.com.cn/; MS, QTRAP®System, https://sciex.com/). Linear ion trap (LIT) MS and triple quadrupole (TQ) MS scans were acquired using a triple TQ-LITmass spectrometer (QTRAP® LC-MS/MS System)that had an ESI Turbo Ion-Spray interfacethatwas operated in positive and negative ion modes, and was controlled by Analyst 1.6.3 software (Sciex).The analytical conditions were as follows, UPLC: column, ThermoAccucore™ C30 (2.6 μm, 2.1 mm*100 mm i.d.); solvent system, A: acetonitrile/water(60/40,V/V, 0.1% formic acid, 10 mmol/L ammonium formate), B: acetonitrile/isopropanol (10/90 V/V, 0.1%formic acid, 10 mmol/L ammonium formate); gradient program, A/B ( 80:20, V/V ) at 0 min, 70:30 V/V at2.0 min, 40:60 V/V at 4 min, 15:85 V/V at 9 min, 10:90 V/V at 14 min, 5:95 V/V at 15.5 min, 5:95 V/Vat 17.3 min, 80:20 V/V at 17.3 min, 80:20 V/V at 20 min; flow rate, 0.35 ml/min; temperature, 45∘C; Injection volume: 2 µl. The effluent was alternatively connected to an ESI-triple quadrupole-linear ion trap(QTRAP)-MS.

### Data processing and statistical analysis

For untargeted metabolomics data, converting the obtained original data into mzXML format (xcms input file format) through Proteowizard(http://proteowizard.sourceforge.net/), using theR package (XCMS) to include peaks identification and peaks filtering filtration, peaks alignmen, and the mass to charge ratio (m/z), retention time. Peak area (intensity) and other data matrix were obtainedfor subsequent analysis. For targeted metabolomics data and MS data were processed using Analystsoftwareversion1.6.3.TheMultiaQuant software was used to open the mass spectrum file of the sample, and the chromatographic peaks were integrated and corrected .All chromatographic peak area integral data were derived and saved. Based on the untargeted and targeted data, 1300 metabolites (320 hydrophilic metabolites and 980 lipophilic metabolites) were retained after elimination of duplicates and quality control. Data were normalized by the sum of theintensity of metabolites, auto-scaled, and then log_2_-transformed for analysis. Missing values were detected and replaced by the half of the minimum value. Multinomial logistic regression models were used to screen for differentially abundant metabolites between different groups. The logistic regression model was: “Group ~ metabolite level + age + sex”. Partial least-squares-discriminant analysis (PLS-DA) was used for assessment of outliers and supervised clustering. Pathway enrichment was performed using MetaboAnalyst version 5.0 (http://metaboanalyst.ca). Statistical analyses were performed using R software version 4.1 and SPSS software version 23.0.

## Results

### General metabolic characteristics of three groups

**Fig. 1:**
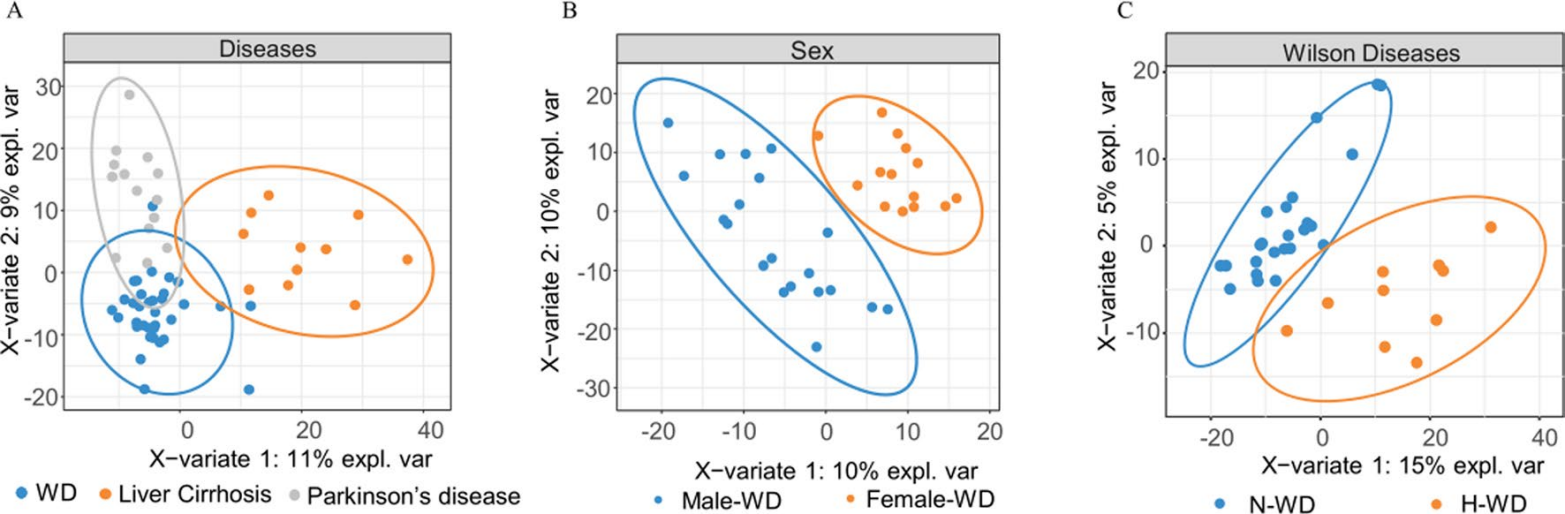
PLS-DA score scatter plots of metabolomicsdatainthe three diseases groups (**A**), two gender group (**B**), and two clinical subtype group (**C**)

To assess the presence of abnormal samples, we performed partial least squares discriminant analysis (PLS-DA) across the three groups. Based on the spatial distributions of the data, we did not observe any notably abnormal samples. Nonetheless the PLS-DA indicated differences in metabolite levels among the WD, liver cirrhosis, and Parkinson’s disease groups(Fig. 1A). Additionally, the PLS-DA outcomes demonstrated substantial variances between genders (Fig. 1B) and between the N-WD and H-WD groups (Fig. 1C).

### Metabolomics in the N-WD and H-WD groups

Overall, 187 metabolites were up-regulated and 107 metabolites were down-regulated in the H-WD group relative to the N-WD group (all *p* < 0.05).

#### Hydrophilic metabolites

**Fig. 2:**
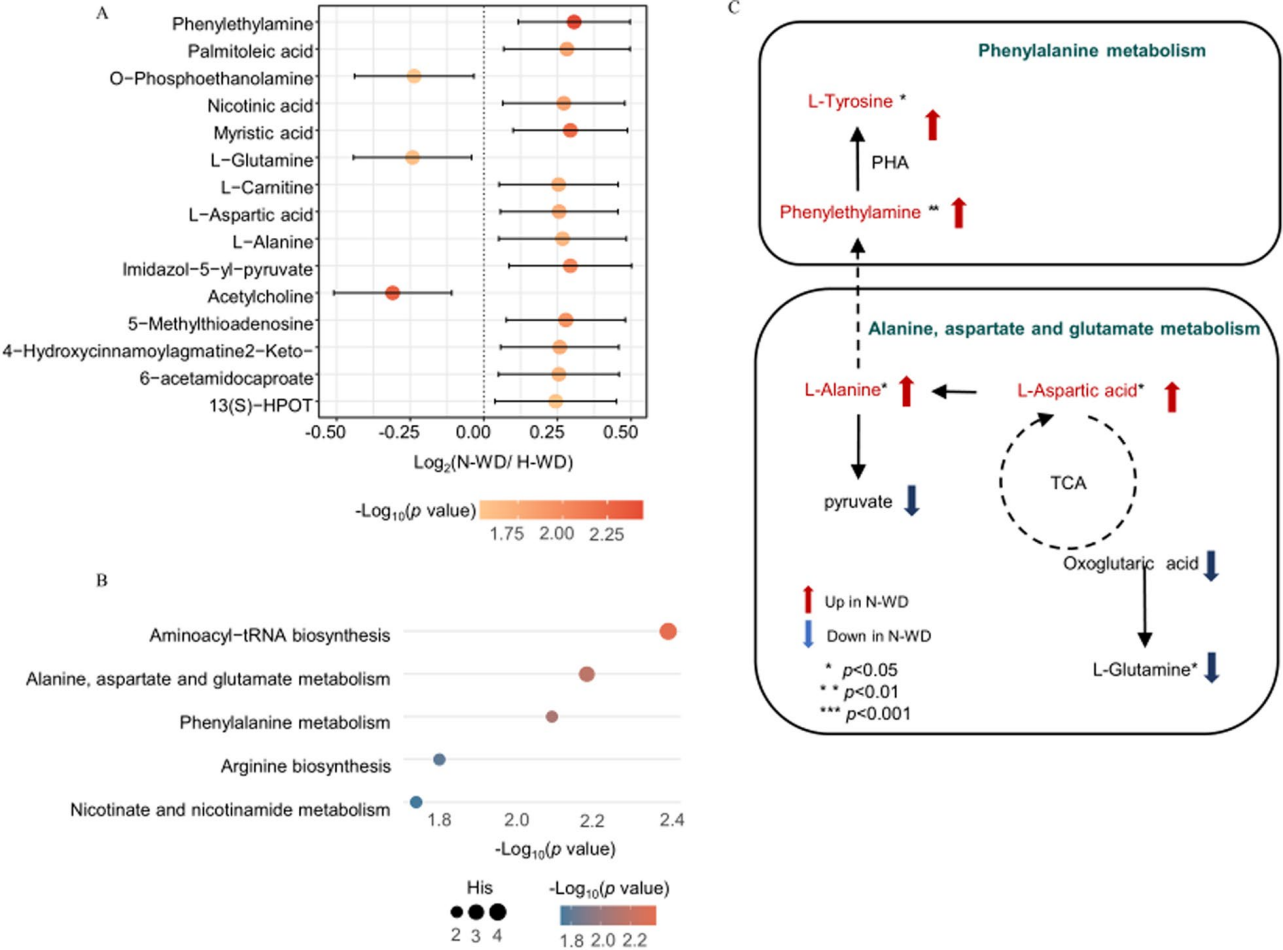
Differences of metabolomic profiles between N-WD and H-WD. **A** Top 15 significant altered hydrophilicmetabolites selected by *p* values. **B** The altered metabolism pathways analyzed by MetaboAnalyst 5.0.Thecolour of each spot was based on the *p*-value, and the size of each spot was based on pathway Hits. **C** An illustration of our metabolomic results and interconnection of main altered pathways

We analyzed the top15 most altered hydrophilic metabolites (Fig. 2A). To identify significantly altered pathways, we employed the pathway analysis module in MetaboAnalyst, which combines hydrophilic metabolite enrichment with pathway topology, using the Kyoto Encyclopedia of Genes and Genomes (KEGG) as reference. The results indicated that the main altered pathways were aminoacyl-tRNA biosynthesis; alanine, aspartate, and glutamate metabolism; phenylalanine metabolism; arginine biosynthesis; and nicotinate and nicotinamide metabolism (Fig. 2B). Of these,the alanine, aspartate, and glutamate metabolism pathway and the phenylalanine metabolism pathway were significantly up-regulated in the H-WD group (Fig. 2C).

#### Lipophilic metabolites

**Fig. 3:**
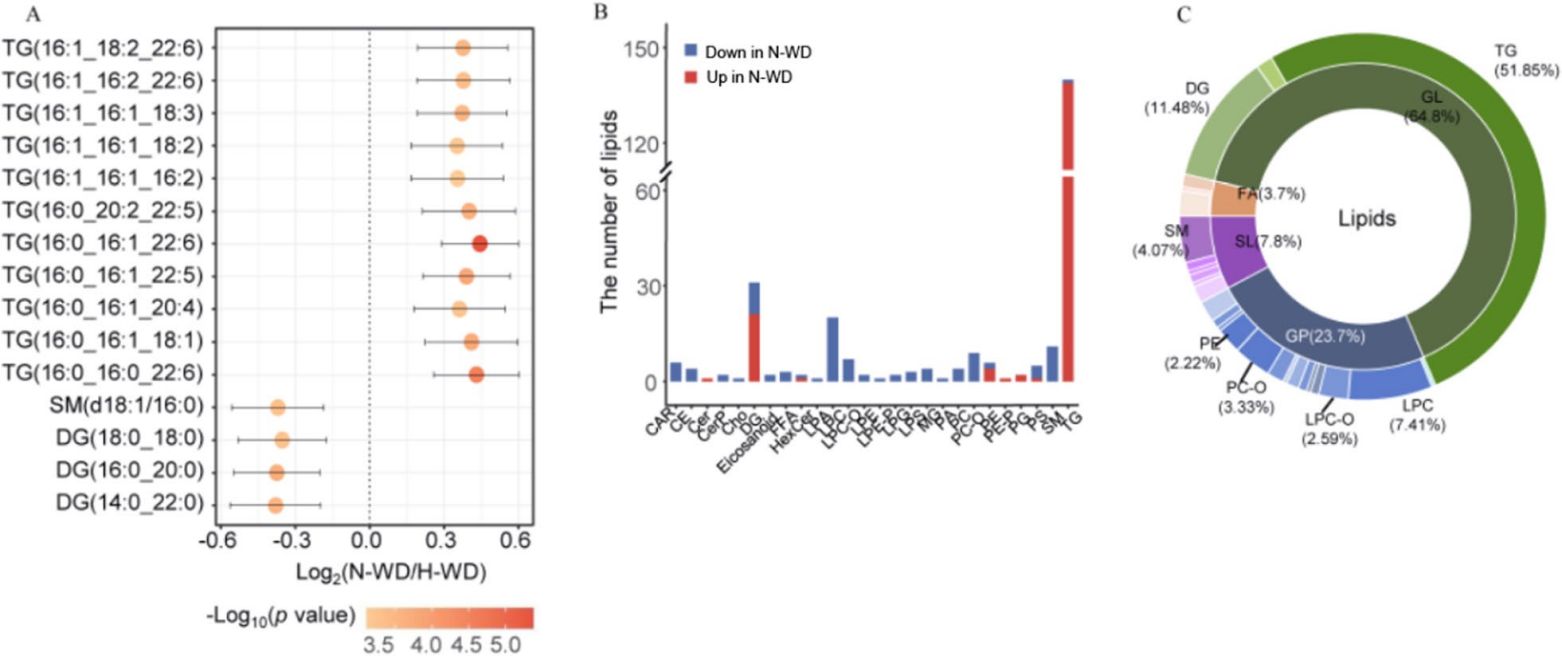
Differences in lipidomic profiles between N-WD and H-WD. **A** Top 15 significant altered lipids selected by *p*values. **B** Differential abundant lipids subclasses screened by multinomial logistic regression. **C** The proportion of differential abundant lipids subclasses

We also identified the top 15 most altered lipophilic metabolites (Fig. 3A). The top three most altered molecules were TG(16:0_16:1_22:6), TG(16:0_16:0_22:6) and TG(16:0_16:1_22:5). Among the significantly altered molecules, the H-WD group had increased levels of Cer, PE-P, PG, and TG, and decreased levels of CAR, CE, CerP, Cho, Eicosanoid, FFA, LPA, LPC, LPC-O, LPE, LPE-P, LPG, LPS, MG, PA, PC, and SM (Fig. 3B). The major differential lipophilic metabolite was TG (51.85%), which was up-regulated in the H-WD group(Fig. 3C).

### Metabolomics in the H-WD and liver cirrhosis groups

Overall, 73 metabolites were up-regulated and 61 metabolites were down-in the H-WB group relative to the liver cirrhosis group (all *p* < 0.05).

#### Hydrophobic metabolites

**Fig. 4:**
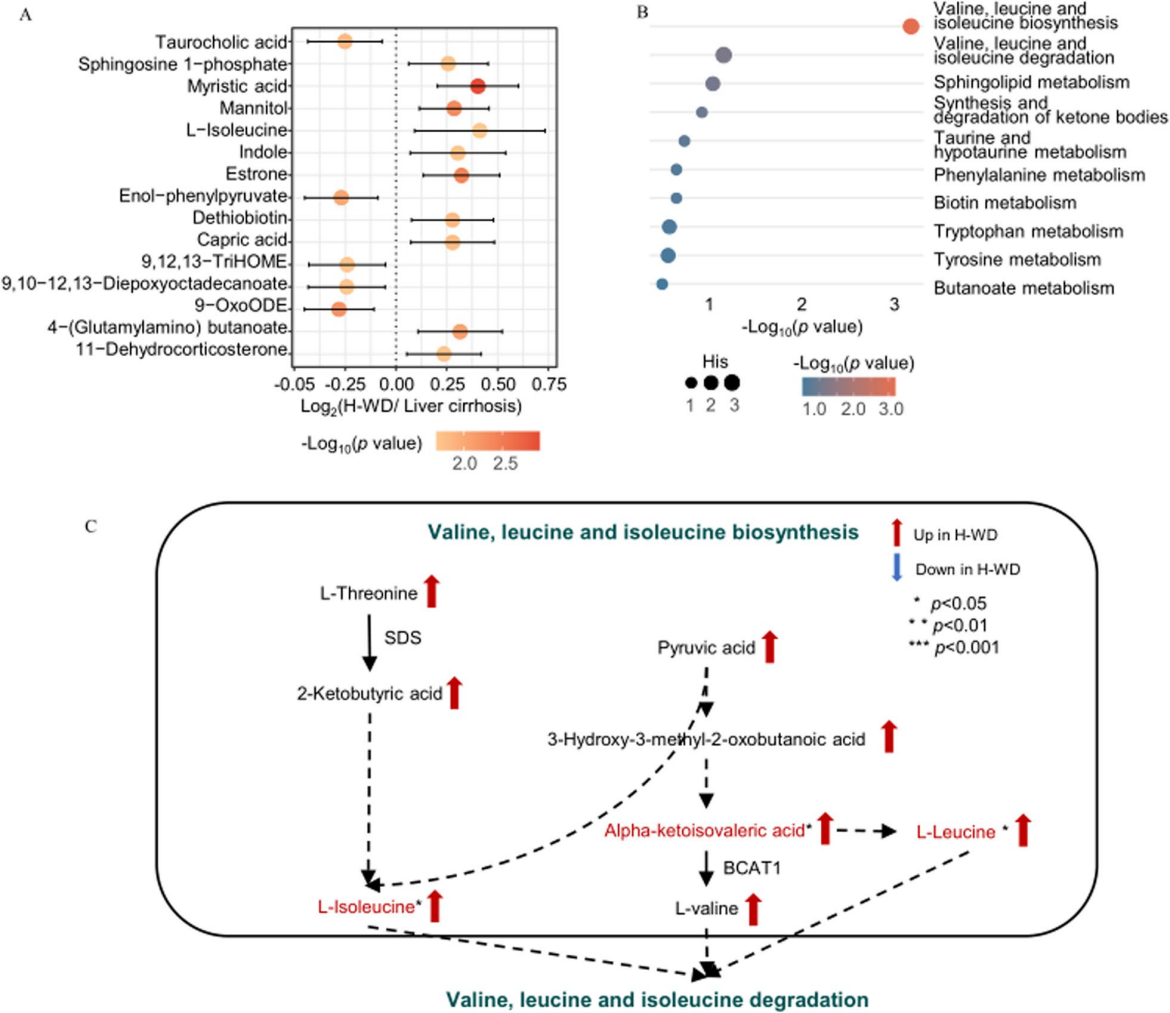
Differences of metabolomic profiles between H-WD and liver cirrhosis. **A** Top 15 significant altered hydrophilicmetabolites selected by p values. **B **The altered metabolism pathways analyzed by MetaboAnalyst 5.0.Thecolour of each spot was based on the p-value, and the size of each spot was based on pathway Hits. **C** An illustration of our metabolomic results and interconnection of main altered pathways

We analyzed the top 15 most altered hydrophilic metabolites (Fig. 4A). As described above, Metaboanalystindicated the most significantly altered metabolic pathway was valine, leucine, and isoleucine biosynthesis, which was significantly upregulated in the H-WD group (Fig. 4B). Biosynthesis mapping using KEGG showed that L-isoleucine, alpha-ketoisovaleric acid, and L-leucine were significantly up-regulated in the H-WD group (Fig. 4C).

#### Lipophilic metabolites

**Fig. 5:**
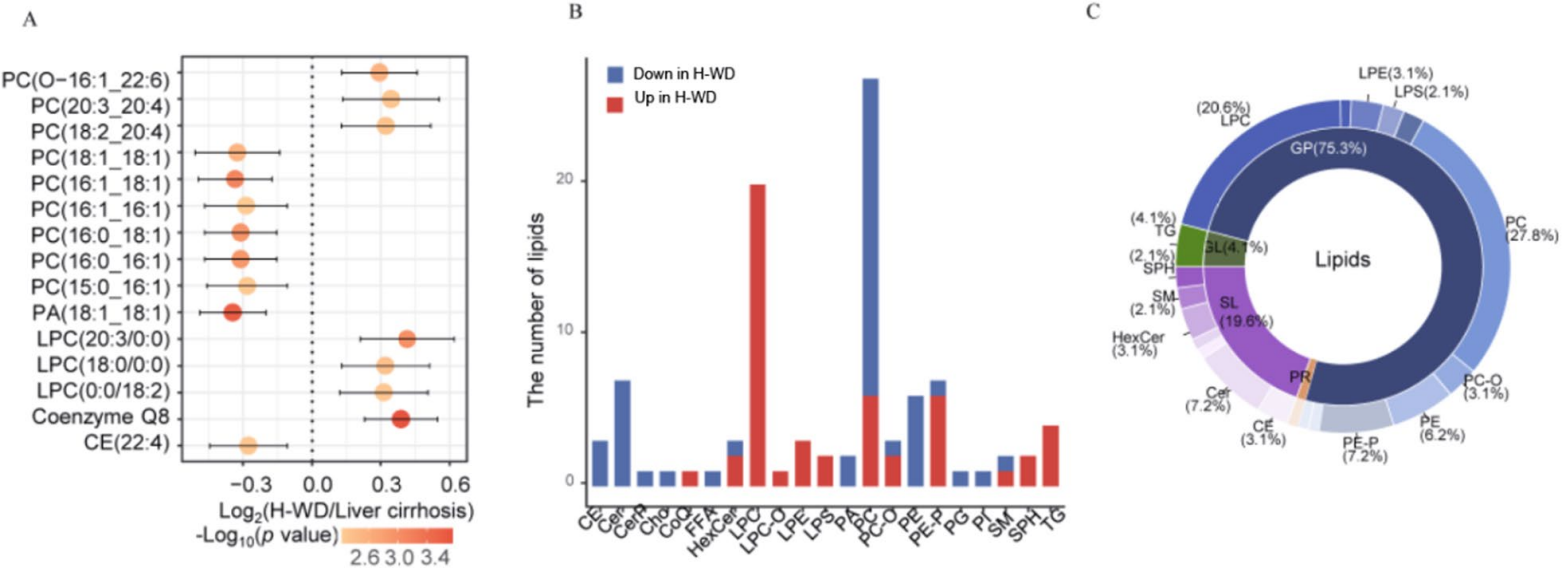
Differences of lipidomic profiles between H-WD and liver cirrhosis. **A** Top 15 significant altered lipids selected by p values. **B** Differential abundant lipids subclasses screened by multinomial logistic regression. **C** The proportion of differential abundant lipids subclasses

We also analyzed the top 15 most altered lipophilic metabolites (Fig. 5A). The top 3 molecules were coenzyme Q8, PA(18:1_18:1), and PC(16:1_18:1). Among the differentially alteredmolecules, CoQ, LPC, LPC-O, LPE, LPS, SPH, and TG were up-regulated, and CE, Cer, CerP, Cho, PA, PE, PG, and PIweredown-regulated in the H-WD group (Fig. 5B). The major differential lipophilic metabolites were PC (27.8%), which was down-regulated in the H-WD group, and LPC(20.6%), which was up-regulated in H-WD group (Fig. 5C).

### Metabolomics in the N-WD and Parkinson’s disease groups

Overall, 116 metabolites were up-regulated and 44 metabolites were down-regulated in the N-WB group relative to the Parkinson’s disease group (all *p* < 0.05).

#### Hydrophobic metabolites

**Fig. 6:**
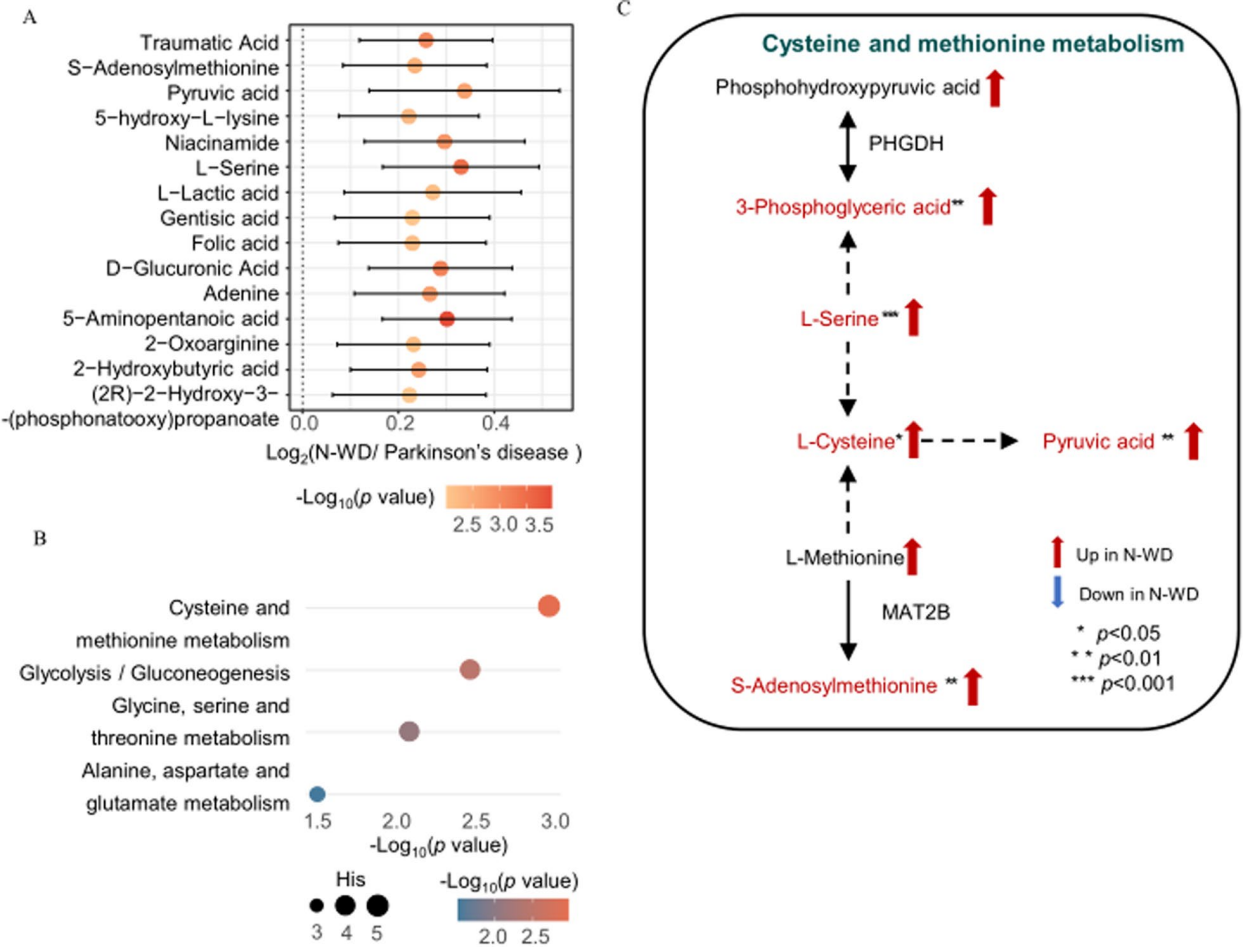
Differences of metabolomic profiles between N-WD and parkinson’s disease. **A** Top 15 significant altered hydrophilic metabolites selected by p values. **B **The altered metabolism pathways analyzed by MetaboAnalyst 5.0. The colour of each spot was based on the p-value, and the size of each spot was based on pathway Hits. **C** An illustration of our metabolomic results and interconnection of main altered pathways

We identified the top 15 most altered hydrophilic metabolites as described above (Fig. 6A). The results from Metaboanalystindicatedthe most significantly altered metabolic pathways were cysteine and methionine metabolism; glycolysis/gluconeogenesis; glycine, serine, and threonine metabolism; and alanine, aspartate, and glutamate metabolism(Fig. 6B). KEGG analysis indicated the altered pathways led to upregulation of 3-phosphoglyceric acid, L-serine, L-cysteine, pyruvic acid, and S-adenosylmethionine in the N-WD group (Fig. 6C).

#### Lipophilic metabolites

**Fig. 7:**
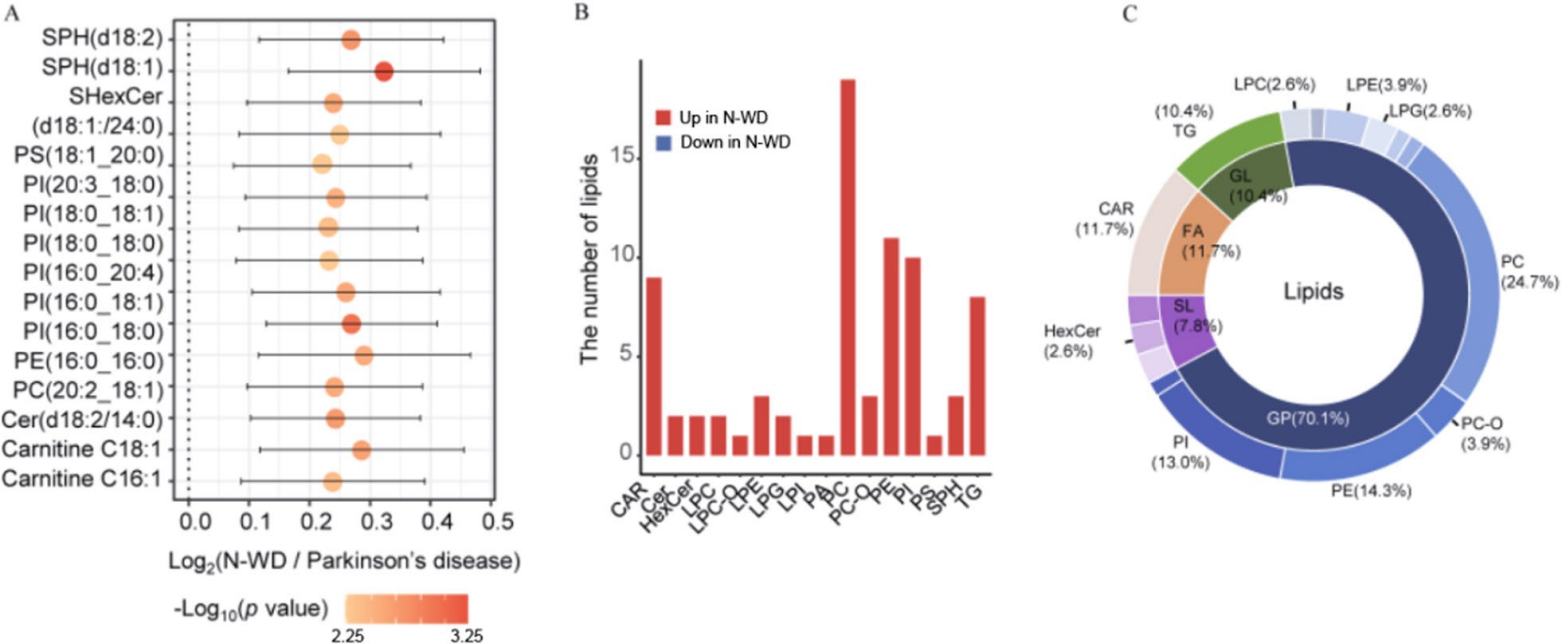
Differences of lipidomic profiles between N-WD and parkinson’s disease. **A** Top 15 significant altered lipids selected by *p* values. **B** Differential abundant lipids subclasses screened by multinomial logistic regression. **C** The proportion of differential abundant lipids subclasses

We identified the top 15 most significantly altered lipophilic metabolites (Fig. 7A). The3 most altered molecules were SPH (d18:1), PI (16:0–18:0), and SPH (d18:2), all of which were up-regulated in the N-WDgroup (Fig. 7B). The molecules with the greatest changes were PC (24.7%) and PE (14.3%) (Fig. 7C).

### Predictive diagnostic model for WD

**Fig. 8:**
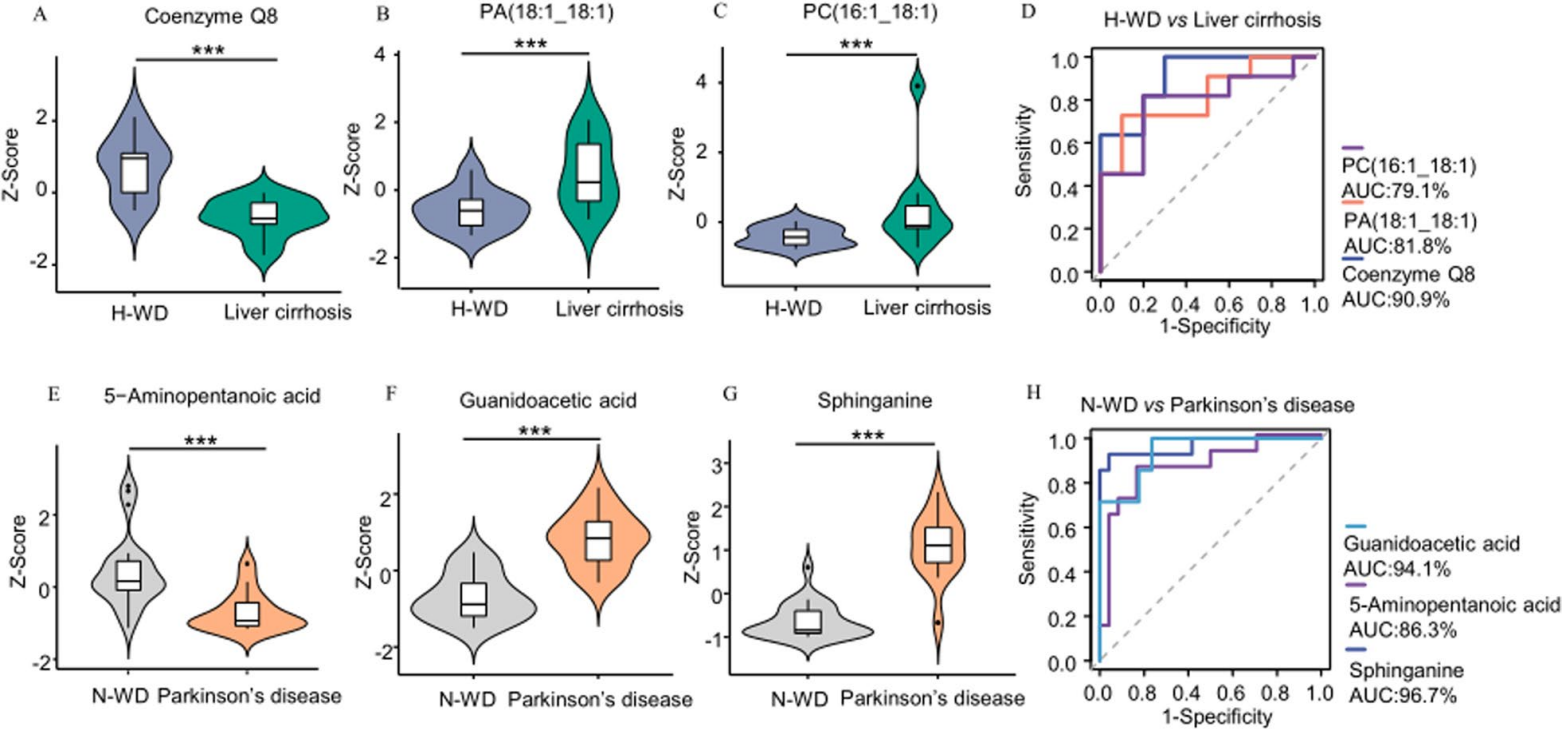
**A**–**C** Relative concentrations of significantly differential abundant metabolites between H-WD andliver cirrhosis. **D** Predictive model for distinguishing H-WD from liver cirrhosis was established by logistic regression. **E**–**G** Relative concentrations of significantly differential abundant metabolites between N-WD and parkinson’s disease. **H** Predictive model for distinguishing N-WD from parkinsons’ disease was established by logistic regression

We then performed receiver operating characteristic (ROC) analysis using the 3 metabolites that had the greatest differences between the H-WD and liver cirrhosis group (Fig. 8A–D) and the greatest differences between the N-WD and Parkinson’s disease group (Fig. 8E–H). In the predictive model of H-WD, the area under the curve (AUC) of coenzyme Q8 was 90.8%, the AUC of PA (18:1_18:1) was 81.8%, and the AUC of PC (16:1_18:1) was 79.1%. In the predictive model of N-WD, the AUC of sphinganine was 96.7%, the AUC of guanidoacetic acid was 94.1%, and the AUC of 5-aminopentanoic acid was 86.3%. Our results indicate that the two groups of metabolites can reliably distinguish H-WD from liver cirrhosis and N-WD from Parkinson’s disease.

## Discussion

WD is a heritable monogenic disorder characterized by abnormal copperaccumulation [4]. Elevated intracellular levels of the copper ionlead to oxidative stress and free radical formation and to mitochondrial dysfunction independent of oxidative stress. The combination of these effects leads to celldeath(cuproptosis) in the liver, brain, and other organs [10, 11]. We first analyzed the metabolic alterations characteristic of WD using PLS-DA. Our results indicated differences between males and females, and between patients with different clinical manifestations (H-WD and N-WD). A previous clinical studies have shown that there are gender differences in Wilson disease [12]. Therefore, we controlled for gender and performed logistic regression screening to identify differences between H-WD and N-WDpatients.Wethen compared the metabolomicandlipidomicprofilesof H-WD and N-WD.

Our metabolomics results showed the valine, leucine, and isoleucine biosynthesis pathways were significantly up-regulated in the H-WD group relative to the N-WD group. In particular, the glutamine level was significantly lower in H-WD group. Glutamine is involved in the synthesis of glutathione (GSH), a key antioxidant, especially in the liver [13]. Thus, the lower level of glutamine in the H-WD group (which may be due to decreased synthesis or increased consumption) may explain the greater liver damage in H-WD patients than N-WD patients [14].The phenylalanine metabolism pathway was significantly down-regulated in N-WD relative to H-WD. Among the hydrophilic metabolites, the levels of phenylethylamine and L-tyrosine were decreased remarkably in the N-WD group. β-phenylethylamineis an endogenous neuroactive trace amine that is widespread in the human central nervous system, and previous research showed that it increased the synaptic levels of dopamine (DA) [15]. L-tyrosine is a precursor of DA, and can effectively increase catecholaminergic activities, including those mediated by DA [16]. Our results and these previous studies suggest that the decreased phenylethylamine and L-tyrosine may responsible for neurological dysfunction in N-WD patients, and may explain the unique neurological manifestations in these patients, such as dysarthria, dystonia, or Parkinsonism. Our lipidomic results showed that TGs were the most altered hydrophobic compounds, and most of TGs were up-regulated in the H-WD group relative to the N-WD group. High TG levels can lead to non-alcoholic fatty liver disease (NAFLD) [17], mitochondrial damage [18], and oxidative stress [19]. Therefore, we speculate that the accumulation of copper ions in the liver and the increased level of TGs lead to oxidative stress in H-WD. In particular, disruption of the electron transport chain could lead to reduced ATP synthesis, and ultimately to liver damage.

The clinical manifestations of liver cirrhosis resemble those of H-WD, and the clinical manifestations of N-WD resemble those of Parkinson’s disease. We therefore performed two pair-wise comparisons to determine the metabolic differences of these different groups. A metabolomic comparison of the H-WD and liver cirrhosis groups showed the metabolic pathways of aminoacyl-tRNA biosynthesis, alanine/aspartate/glutamate metabolism, phenylalanine metabolism, arginine biosynthesis, and nicotinate/nicotinamide metabolism were significantly up-regulated in the H-WD group. In the valine, leucine, and isoleucine biosynthetic pathway,L-isoleucine, L-leucine, and α-ketoisovaleric acid had the greatest differences among hydrophilic metabolites, and these metabolites were all significantly increased in the H-WD group relative to the liver cirrhosis group. Our analysis of the lipidomic profiles indicated the hydrophobic compound with the major difference was PC, which was up-regulated in the liver cirrhosis group.

Our metabolomic comparison of the N-WD and Parkinson’s disease groups indicated the most altered metabolic pathways were cysteine/methionine metabolism, glycolysis/gluconeogenesis, glycine/serine/threonine metabolism, and alanine/ aspartate/glutamate metabolism. Our comparison of the lipidomic profiles also showed that the major altered hydrophobic compound was PC, and all differential lipids were up-regulated in the N-WD group.

WD is diagnosed based on clinical and laboratory examinations, and a low level of ceruloplasmin, increased urinary level of copper excretion, increased hepatic level of copper, and copper deposition in the cornea (Kayser-Fleischer ring) are common in patients with WD [20]. However, these indicators do not have high diagnostic specificity [21]. We therefore used the novel approach of metabolomics to distinguish WD from analogous diseases(liver cirrhosis and Parkinson’s disease). The results of our logistic regression and ROC analysis indicated that 3 metabolites reliably differentiated H-WD from liver cirrhosis, and 3 other metabolites reliably differentiated N-WD from Parkinson’s disease. The individual AUC values of these metabolites ranged from 79.1 to 90.8% for H-WD,and from 86.3 to 96.7% for N-WD. Thus, these metabolites have good predictive value and their use in clinical practice may provide important diagnostic guidance.

Although our results were statistically and clinically significant, three limitations in this study must be mentioned. The first is insufficient sample size, although this was somewhat unavoidable because of the rarity of WD. Secondly, we did not identify a common crucial feature shared by these diseases. Comparison of the metabolic profiles between different groups may have masked important features (metabolites and pathways) which are common to both pathologies. In addition, we only analyzed metabolites and pathways that had the greatest differences between groups. Other metabolomic differences between groups may also have potential value, a topic that needs further study.

## Conclusion

Our study of the serum metabolomic profiles of patients with WD revealed noteworthy distinctions between genders, as well as between patients afflicted with the H-WD and N-WD subtypes. Although the overt clinical symptoms are similar in H-WD and liver cirrhosis patients, and in N-WD and Parkinson’s disease patients, the metabolic profiles of H-WD and N-WD patients are unique. Our identification of metabolites with the most significant differences and our pathway analysis led to establishment of predictive models for diagnosis of WD. These models identified potential diagnostic biomarkers for WD that may be useful in the future.

## Data Availability

Not applicable.

## Abbreviations

FA: fatty acyl
BA: bile acid
FFA: free fatty acid
CAR: acyl carnitine
GL: glycosphingolipids
DG: diglyceride
MG: monoglyceride
TG: triglyceride
GP: glycerophospholipid
LPC: lysophosphatidylcholine
LPG: lysophosphatidylglycerol
LPI: lysophosphatidylinositol
PC: phosphatidylcholine
PE: phosphatidylethanolamine
PG: phosphatidylglycerol
PI: phosphatidylinositol
PS: phosphatidylserine
LPA: lysophosphatidic acid
PA: phosphatidic acid
LPC-O: LPCwith alkyl and alkenyl substituents
LPE: lysophosphatidylethanolamine
LPE-P: lysophosphatidyl ethanolamine with alkenyl substituents
LPS: lysophosphatidylserine
PC: phosphatidylcholine
PC-O: PC with alkyl and alkenyl substituents
PR: prenollipids
CoQ: coenzyme Q
SL: sphingolipids
CerP: ceramides phosphate
Cho: cholesterol
CE: cholesterol Ester
SPH: sphingosine
Cer: ceramide
HexCer: hexosylceramides
SM: sphingomyelin
